# Making sense of cardiomyopathy caused by deletion of acetyl-CoA carboxylase 1 and 2. Reply.

**DOI:** 10.1172/JCI210573

**Published:** 2026-09-01

**Authors:** Chai-Wan Kim, Matthew A. Mitsche, Jay D. Horton

**Affiliations:** 1 Center for Human Nutrition,; 2 Department of Internal Medicine, and; 3 Department of Molecular Genetics, University of Texas Southwestern Medical Center, Dallas, Texas, USA.

**Keywords:** Cardiology, Metabolism, Fatty acid oxidation, Heart failure

**The authors reply:** We appreciate the Letter by Tian and Lewandowski highlighting the distinct canonical functions of acetyl-CoA carboxylase 1 (ACC1) and ACC2 and the metabolic complexity of our combined deletion model (1). Their Letter questions whether the relatively modest increase in fatty acid oxidation (FAO) observed in ACC1/2 deletion (dHKO) hearts is sufficient to explain the severe cardiomyopathy. Although the cardiomyopathy in ACC1/2 dHKO mice may not be exclusively attributable to increased FAO, prevention of heart failure by two pharmacologic FAO inhibitors, etomoxir and oxfenicine, strongly supports a primary causal role for increased FAO.

The prior cardiomyocyte-specific ACC2 HKO study reported preserved cardiac function for up to 12 months (2). We do not view this as contradictory. ACC2 loss alone may be tolerated when compensatory lipid-homeostatic mechanisms remain intact, whereas additional loss of ACC1-dependent lipid homeostasis may lower the threshold for decompensation.

The severity gradient of cardiomyopathy in our study directly addresses this issue (3): ACC1/2 dHKO mice were most severely affected, ACC2 HKO mice developed milder progressive dysfunction, and ACC1 HKO mice remained closest to controls. This pattern argues that ACC1 loss alone does not explain the early and severe cardiomyopathy in dHKO mice and is consistent with a major contribution from ACC2 loss and increased mitochondrial fatty acid entry.

Tian and Lewandowski note that FAO and the FA/glucose oxidation ratio increased less in ACC1/2 dHKO hearts (~15% and 2-fold) than in ACC2 HKO hearts (~50% and >3-fold), although the prior ACC2 HKO model did not develop cardiomyopathy (1). Cross-study comparisons of FAO magnitude are difficult to interpret in isolation. ACC2 HKO hearts retained approximately 50% of total malonyl-CoA and were protected against pressure overload–induced dysfunction, whereas malonyl-CoA was reduced by approximately 89% in ACC1/2 dHKO hearts, and they developed a cardiomyopathy at 8 weeks of age. The models therefore differ in residual malonyl-CoA, physiological context, duration of metabolic stress, and capacity to maintain membrane lipid homeostasis. These factors, rather than FAO measured at a single time point, may determine whether increased FAO is adaptive or pathogenic.

The genotype-dependent severity gradient also addresses the Myh6-Cre toxicity concern (4). ACC1 HKO mice generated with the same Cre driver provide an internal reference for Cre-associated effects; the greater impairment in ACC2 HKO and dHKO mice cannot be explained by Cre alone. Additionally, the prior ACC2 HKO study used an αMHC/Myh6-Cre driver (2), so Cre-related concerns should apply equally.

Disease onset coincides with postnatal cardiac metabolic maturation, when cardiomyocytes increase reliance on mitochondrial FAO (5). This timing does not necessarily implicate ACC1 loss as the primary driver; rather, it may reflect heightened dependence on ACC2-mediated restraint of mitochondrial fatty acid entry during this vulnerable transition. Etomoxir initiated at 4 weeks prevented dysfunction, whereas treatment initiated at 20 weeks did not reverse established cardiomyopathy. Although Tian and Lewandowski suggest that adult treatment trended toward worsening, differences in ejection fraction and fractional shortening were not significant, and the left ventricular mass change may reflect disease progression rather than drug toxicity.

Tian and Lewandowski also note that dietary linoleic acid failed to rescue the dHKO phenotype. We did not claim to have directly demonstrated selective oxidation of linoleic acid. dHKO hearts showed broad fatty acid depletion, with linoleic acid showing the greatest reduction. Uptake of linoleic and oleic acids was preserved, yet labeled linoleate incorporation into phosphatidylcholine (PC) and cardiolipin was reduced, whereas labeled oleate incorporation was unchanged. FAO inhibition restored linoleoyl phospholipids and cardiolipin. Because linoleoyl-PC and linoleoyl-phosphatidylethanolamine are acyl donors for cardiolipin remodeling, their depletion provides a mechanistic link to loss of tetralinoleoyl cardiolipin. Dietary supplementation raised cardiac linoleate without restoring it to WT levels, which may explain the lack of functional rescue.

We agree that ACC1 may contribute to cardiomyocyte lipid homeostasis. However, because ACC1 HKO hearts remained closest to controls, ACC1 loss alone appears insufficient to explain the early and severe dHKO phenotype.

In summary, our data support a model in which loss of ACC2-dependent restraint of FAO is a major disease determinant, while ACC1 loss lowers the threshold for cardiolipin depletion and mitochondrial dysfunction. Increased FAO may be tolerated when compensatory mechanisms are sufficient but becomes maladaptive when those mechanisms are compromised.

## Version 1 09/01/2026

Electronic publication
